# Very High Cycle Fatigue Behavior of a Directionally Solidified Ni-Base Superalloy DZ4

**DOI:** 10.3390/ma11010098

**Published:** 2018-01-10

**Authors:** Baohua Nie, Zihua Zhao, Shu Liu, Dongchu Chen, Yongzhong Ouyang, Zhudong Hu, Touwen Fan, Haibo Sun

**Affiliations:** 1 School of Materials Science and Energy Engineering, Foshan University, Foshan 528000, China; niebaohua121@163.com (B.N.); liushu814194678@126.com (S.L.); chendc@fosu.edu.cn (D.C.); zdhu@live.com (Z.H.); fantouwen_1980@163.com (T.F.); sunmyseven@126.com (H.S.); 2 School of Materials Science and Engineering, Beihang University, Beijing 100191, China; 3 School of Environmental and Chemical Engineering, Foshan University, Foshan 528000, China; ouyang7492@163.com

**Keywords:** DZ4, casting defects, VHCF, crack initiation

## Abstract

The effect of casting pores on the very high cycle fatigue (VHCF) behavior of a directionally solidified (DS) Ni-base superalloy DZ4 is investigated. Casting and hot isostatic pressing (HIP) specimens were subjected to very high cycle fatigue loading in an ambient atmosphere. The results demonstrated that the continuously descending S-N curves were exhibited for both the casting and HIP specimens. Due to the elimination of the casting pores, the HIP samples had better fatigue properties than the casting samples. The subsurface crack initiated from the casting pore in the casting specimens at low stress amplitudes, whereas fatigue crack initiated from crystallographic facet decohesion for the HIP specimens. When considering the casting pores as initial cracks, there exists a critical stress intensity threshold ranged from 1.1 to 1.3 $\mathrm{MPa}\sqrt{m}$, below which fatigue cracks may not initiate from the casting pores. Furthermore, the effect of the casting pores on the fatigue limit is estimated based on a modified El Haddad model, which is in good agreement with the experimental results. Fatigue life for both the casting and HIP specimens is well predicted using the Fatigue Indicator Parameter (FIP) model.

## 1. Introduction

Directionally solidified (DS) Ni-based superalloys are served as aero-engines turbine blades due to their exceptional high resistance to creep, oxidation, and corrosion, as well as good fracture toughness. Low cycle fatigue (LCF) and thermomechanical fatigue behaviours [1,2] of the alloys have been well studied, because they were believed to be significant during start-up and shut down of engines. However, in the actual service environment of turbine blades, blade excitation frequencies exceed 1 kHz due to the transient airflow dynamics, the processes of which are usually simulated as very high cycle fatigue (VHCF) damage [3]. However, the VHCF behavior of the directional solidified Ni-base superalloys has not been well investigated so far. On the other hand, DS superalloys often contain casting pores, microshrinkages, and other inhomogeneities, which significantly reduce the fatigue properties. The size and the distribution of the defects vary substantially for different casting samples, resulting in the large scatter of the fatigue data, which is remarkably higher in very high cycle fatigue region. It obviously limits the application of this alloy as cyclically loaded components for which VHCF fatigue strength is required.

Though the casting porosity is relatively small, fatigue crack is predominately initiated from the internal casting pore in VHCF region. VHCF crack initiation from the internal casting pore and the subsequent planar slip propagation were observed in Ni-based single crystal superalloys [3,4]. Cyclic stress leads to the formation of the persistent slip bands running through the γ matrix and the γ’ precipitates, which represents sites for the initiation (in interaction with casting pores and other defects) [4]. The development of crystallographic planes is promoted by the stress concentration effect of casting defects [5]. However, some small casting defects are irrelevant to the fatigue crack initiation, and consequently to the fatigue life [5]. Thus, the effect of casting defects on very high cycle fatigue properties and fracture mechanism is not well understood in DS Ni-base superalloys. 

In the past years, a Kitagawa–Takahashi (K–T) diagram [6] joined by the El Haddad model was proved to be useful in evaluating the potential for small cracks to reduce the HCF capability of a material [7]. Morrissey [8] investigated the effect of LCF on HCF limits based on the K–T diagram with LCF crack depth. When considering Murakami’s concepts [9], an El-Haddad model based on the root-area parameter was adopt by Beretta [10], which also indicated how ∆K_th_ increases with the size of the defect (crack). Recently, Zerbst [11] modified the El Haddad model based on the cyclic R curve, which can be described by giving the fatigue crack propagation threshold as a function of the crack extension. As compared with HCF, VHCF has lower fatigue limits at 10^9^ cycles, and has lower tolerance to materials defects. It is expected that the materials defects can significantly affect the VHCF properties, which can be quantitatively investigated based on these modified El-Haddad models. 

For practical application of the casting superalloys, hot isostatic pressing (HIP) is introduced to reduce or eliminate casting defects (such as casting pores, cavity shrinkage), which can result in the increase of the fatigue properties for the materials and the narrowing of the scatter of fatigue data in comparison with the casting materials [12]. In the present study, very high cycle fatigue behavior of the casting DZ4 alloys, which was developed to be used in gas turbine engines due to the low cost, were investigated in comparison with the HIP ones. It is expected to quantitatively evaluate the influence of the casting defects on fatigue properties, as well as to clarify the crack initiation mechanism.

## 2. Experimental Procedures

### 2.1. Materials

**Figure 1:**
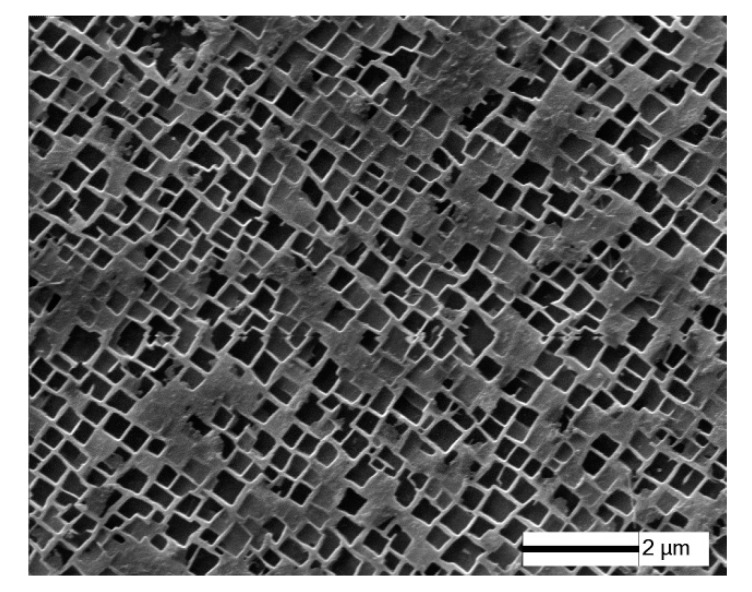
Microstructure of as heat-treated directionally solidified DZ4 Ni-base superalloy.

The nominal composition of the DZ4 alloy used in this study is listed in Table 1. The alloy is characterized by small cobalt content, excluding Hf. The DS specimens were made by the process of high rate solidification in a vacuum induction directional solidification furnace (SKY Technology Development Co., Ltd., Shenyang, China). The procedure of heat treatment was following: 1220 °C/4 h AC, 870 °C/32 h AC (AC: air cooling). The process of the heat treatment produced a precipitation of regular cuboidal γ’ particles aligning along [001] distributed in the matrix (Figure 1). The size of γ’ was about 0.3 μm and the volume fraction of γ’ phase was about 72%. The heat-treated material obtained a high yield strength of 947 MPa and tensile strength of 1059 MPa.

Some specimens were processed by HIP to eliminate the casting defects. The finally machined specimens were heated at 1180 °C at a pressure of 1500 bar for 3 h. The cooling rate was 10 °C/min and the process was performed under Ar atmosphere down the 900 °C, later on the cooling was conducted on air. The specimens were treated following: 1220 °C/4 h AC, and 870 °C/32 h AC. 

### 2.2. Surface Treatment

The specimens underwent electro-polishing (EP) to remove the machining layers that could affect the fatigue behavior of the samples. Electro-polishing was carried out in 10% perchloric acid and 90% ethanol under −20 °C and 20–25 V voltage. 

### 2.3. Ultrasonic Fatigue Test

Fatigue tests were carried out using an ultrasonic fatigue test machine (20 kHz, SHIMADZU, Kyoto, Japan) at a constant load ratio of R = −1. This method is an accelerated testing method with a frequency far beyond that of the conventional fatigue experiments [13]. This method is more effectiveness than the conventional tests method. The frequency effect is small for ultrasonic fatigue tests under low displacement and small deformation [14], and very high cycle fatigue behaviors of many metallic materials have been investigated using an ultrasonic fatigue test machine, which includes an ultrasonic generator, a piezoelectric converter, an ultrasonic horn and a computer control system [13]. An ultrasonic generator transforms 50 or 60 Hz voltage signal into sinusoidal signal with 20 kHz; a piezoelectric converter excited by the generator transforms the electrical signal into longitudinal mechanical vibration with same frequency; an ultrasonic horn amplifies the vibration displacement in order to obtain the required strain amplitude in the middle section of specimen; a computer control system is necessary to control the load amplitude and acquire test data. The maximum displacement amplitude measured by means of a dynamic sensor is obtained at the end of the specimen, while the strain excitation in push–pull cycles (load ratio R = −1) reaches the maximum in the middle section of the specimen, which produces the required high frequency fatigue stress. In addition, a compressed air cooling gun is necessary to be used to prevent the temperature increasing of specimen in the tests.

**Figure 2:**
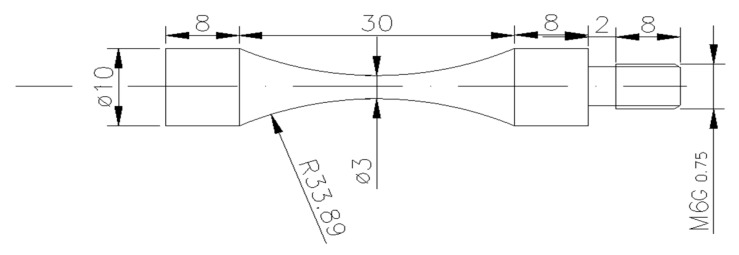
Shape and dimensions of the test specimens.

When considering that the amplifier and the specimen must work at resonance, the specimen geometry was designed using the elastic wave theory. Figure 2 shows the geometries of the fatigue specimens and its dimensions.

## 3. Results

### 3.1. VHCF Properties

**Figure 3:**
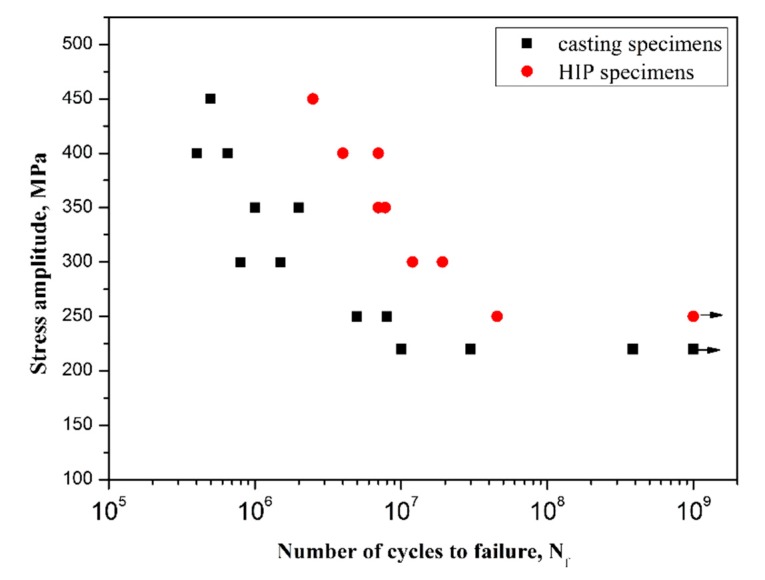
S-N curves of casting and hot isostatic pressing (HIP) specimens (Arrows denote the run-out specimens).

The VHCF fatigue life for both the casting and the HIP specimens are shown in Figure 3. It is shown that the continuously descending S-N curves are exhibited in both specimens, and there is a knee of horizontal lines in the regime above 10^7^ cycles, where the S-N data exhibit substantial scatter at fatigue limits for both conditions. Fatigue fracture still occurs in both casting and HIP specimens when the number of cycles is larger than 10^7^ cycles. The shift makes more than 50 MPa for a lifetime of 10^7^ cycles to failure in HIP specimens. It is demonstrated that HIP treatment can significantly improve the fatigue properties. At the same applied stress amplitudes, the HIP specimens exhibited a longer fatigue life than the casting ones, which is consistent with Kun’s work for HCF properties [12]. However, Fatigue limit of the HIP specimens is increased from 220 to 250 MPa when compared with the casting specimens. 

### 3.2. Fractograph

**Figure 4:**
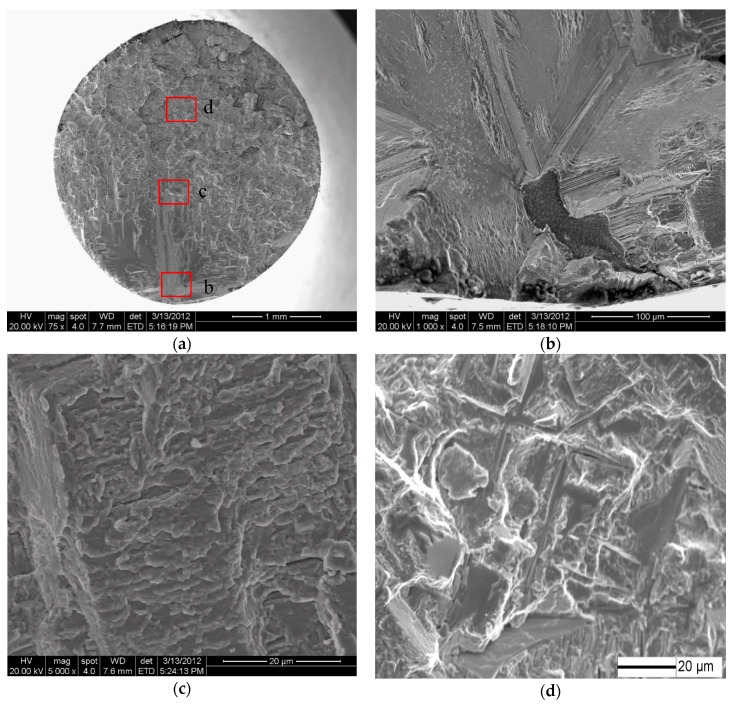
SEM (scanning electron microscopy) images of fracture surface of casting specimens fatigue tested at σ_a_ = 400 MPa and N_f_ = 6.94 × 10^5^. (**a**) Overview of fracture surface; (**b**) magnification micrograph of crack initiation region; (**c**) step-like feature in the crack propagation region; and, (**d**) fatigue fracture zone. Figure 4b–d are indicated in Figure 4a.

**Figure 5:**
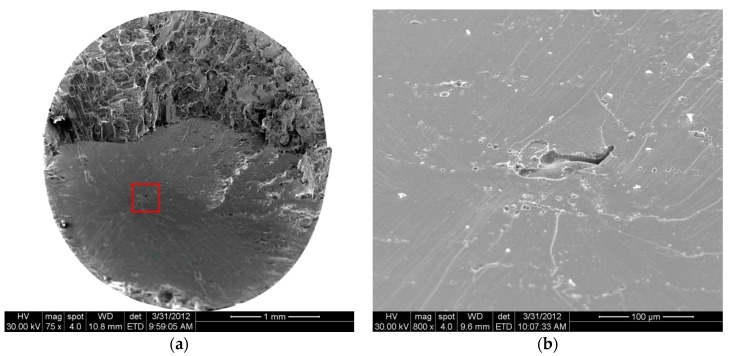
SEM images of fracture surface of casting specimens fatigue tested at σ_a_ = 220 MPa and N_f_ = 3.84 × 10^8^. (**a**) Overview of fracture surface; (**b**) magnification micrograph of crack initiation region as indicated by frame in Figure 5a.

As for the casting specimens, scanning electron microscopy (SEM) observation shows that the fatigue crack initiates from the inner casting pore in the range from 10^5^ to 10^9^ cycles. The fracture mode exhibits strong stress amplitude dependence. Figure 4 shows typical SEM images of the fracture surface at 400 MPa. Crack initiated at the inner casting pore and propagated along a defined direction, which can be characterized as stage I, propagation along cleavage facet (Figure 4b), followed by stage II, cracking perpendicular to the applied stress direction. At stage I, the fracture surfaces is occupied by plane featured facets connected with ridges, which is brought about by the simultaneous slip on different slip planes. At the end of the crack propagation stage, the stepwise feature on the fracture surface can be seen in Figure 4c. One stair formed within one cycle but one cycle unnecessarily induced the formation of one stair. The dendrite structure can be observed in the fatigue fracture zone (Figure 4d). However, only stage I crack propagation can be observed in very high cycle regimes at low stress amplitude (Figure 5). The crack initiated at the inner casting pore and propagated along cleavage facet (Figure 5b). The presence of slip traces on the fracture surfaces provided another evidence for cyclic plastic deformation. The facets ridges and stepwise feature is not observed.

**Figure 6:**
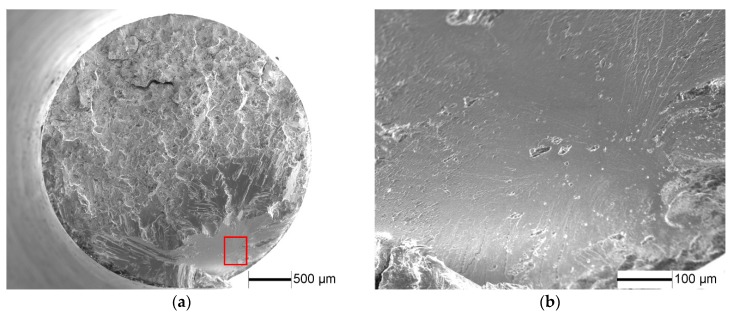
SEM images of fracture surface of HIP specimens fatigue tested at σ_a_ = 250 MPa and N_f_ = 4.53 × 10^7^ cycles. (**a**) Overview of fracture surface; (**b**) magnification micrograph of crack initiation region as indicated by frame in Figure 6a.

As for the HIP specimens, the fracture modes are similar to, but not the same as, those of the casting specimens. Figure 6 shows typical SEM images of fracture surface for the HIP specimen at 250 MPa stress amplitude. It can be seen that the cracks initiate at the inner of specimen (Figure 6b), and a short stage I crack propagation is followed by a dominant stage II crack propagation. However, the crack do not initiates from the small pore that can be eliminated by HIP treatment, indicating the small pore has no influence on the crack initiation.

## 4. Discussion

### 4.1. Effect of Casting Pore on Fracture Mechanism

In Ni-base superalloys, the fracture surface is typically covered by wide plane facets lying along {111} crystallographic planes. Heterogeneous cyclic slip activity occurs on the {111} planes up to the grain size, highly inhomogeneous dislocation arrangement parallel to the {111} planes passes through the γ matrix and small γ’ precipitate [6]. These slip bands are weakened planes, and the decohesion along {111} planes can take place due to the cycling, which can be considered as the formation of the internal cracks. The surface of facets corresponding to the fine γ/γ’ structure is very smooth. In some cases, these plane facets enclose the casting defect; it seems to be natural that the stress concentration effect of casting defects promotes the development of these slip bands (Figure 4 and Figure 5). On the other hand, small casting defects are irrelevant to the fatigue crack initiation, and consequently to the fatigue life (Figure 6). The slip band activity in suitably orientation can also take place without any stress concentration effect of the casting defects. In this case, the most decisive factor induced the crack initiation seems to be the slip activity. 

**Figure 7:**
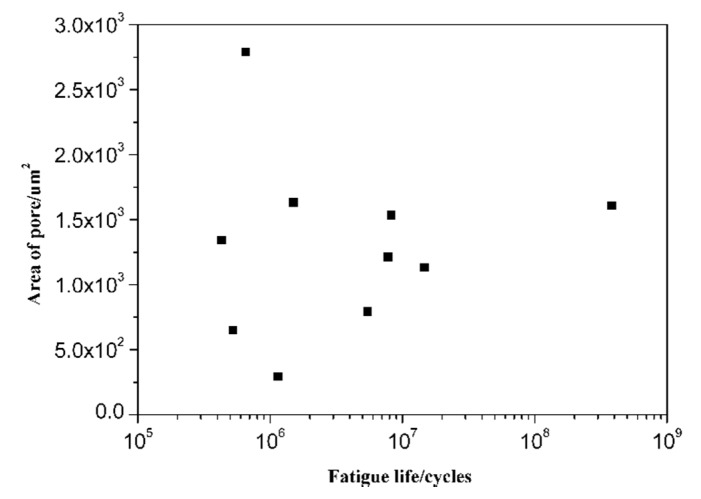
Relationship between the size of casting pore and fatigue life.

As for the casting specimens, the casting defects have significantly influenced the fatigue crack initiation. Figure 7 shows the experimental relationship between the area of the casting pore at the internal crack origin and the number of cycles to failure (N_f_). The area of the casting pore range from 290 to 2790 µm^2^. It is indicated that the area of the casting pore is independent of fatigue life N_f_. 

For the internal fracture model, the initial stress intensity factor (SIF) range at the front of the casting pore is calculated using the following formula [9]:

(1) $$\Delta K_{pore}=0.5\sigma_{a}\sqrt{\pi \sqrt{{\mathrm{area}}_{pore}}}$$

where σ_a_ is the stress amplitude and area*_pore_* is the area of the casting pore at the crack origin. In ultrasonic fatigue with a mean load equal to zero (R = −1), only the tensile part of the cycle has a predominant effect on the fatigue crack propagation [15]. Thus, Δ*K_pore_* could be calculated by substituting σ_a_ into Equation (1), instead of Δσ. 

**Figure 8:**
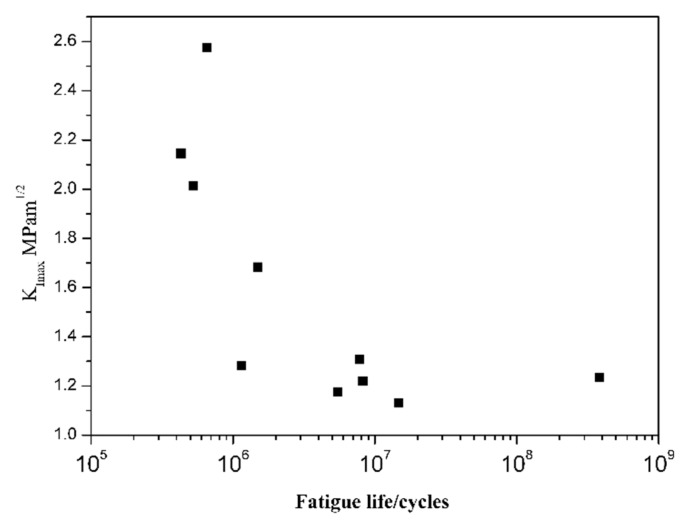
Relationship Δ*K_pore_* with fatigue life.

Figure 8 shows that Δ*K_pore_* gradually decreases with increasing fatigue life for 10^5^–10^7^ cycles, whereas Δ*K_pore_* has a constant trend (1.1–1.3 $\mathrm{MPa}\sqrt{m}$) in the range from 10^7^ to 10^9^ cycles. It is indicated that fatigue cracks, where stress intensity factor is higher than the constant Δ*K_pore_*, can initiate at pores, and propagate until failure. Mayer [16] suggested that there exists a critical stress intensity factor, Δ*K_cr_*, for casting alloys, below which fatigue crack can initiate at the casting pore, but cannot propagate to failure. Thus, the constant Δ*K_pore_* can be considered as a Δ*K_cr_* of the casting specimens. When the SIF of the casting pore is lower than its threshold, fatigue crack may initiate from the casting defects, but may not propagate to fracture, or the crack can initiate from other materials defects, such as persist slip, grain boundary [17]. As for HIP specimens, which pores did not affect the fatigue crack initiation, fatigue cracks initiated from slip plane and propagated along them (Figure 6).

### 4.2. Effect of Casting Pore on Fatigue Limits

According to Murakami’s concepts [9], fatigue limit in the presence of defects corresponds to the threshold condition of non-propagating cracks emanating from the defects themselves, which can, thus, be treated as small cracks. Zerbst [11] modified the El Haddad model based on the cyclic R curve, which can be described by giving the fatigue crack propagation threshold as a function of the crack extension. When considering the internal casting pore, the parameter of the crack depth crack can be replaced by the root-area parameter [10], which can substitute for the crack depth in Zerbst’s modified El Haddad model [11], thus the endurance limits of DZ4 alloy contained casting pores is obtained as:

(2) $$\Delta \sigma =\frac{\Delta K_{th}(\sqrt{area_{defect}})}{Y\sqrt{\pi (\sqrt{area_{i}}+\sqrt{area_{defect}})}}$$

which is the mathematical description of the K–T diagram. Δσ is replaced by stress amplitude $\sigma_{a}$. The geometry factor *Y* is equal to 0.5 for internal casting pores. $\Delta K_{th}^{}(\sqrt{area_{defect}})$ is the threshold of stress intensity factor for small materials defect, $\sqrt{area_{i}}$ and $\sqrt{area_{defect}}$ are the initial closure-free equivalent crack size and the materials defects, and fatigue crack $\sqrt{area}=\sqrt{area_{i}}+\sqrt{area_{defect}}$.

El Haddad’s $\Delta K_{th}^{}(\sqrt{area_{defect}})$ equation has to be modified by adding an additional term $\sqrt{area^{*}}$ [11]:

(3) $$\Delta K_{th}^{}(\sqrt{area_{defect}})=\Delta K_{th,LC}\sqrt{\frac{\sqrt{area_{defect}}+\sqrt{area^{*}}}{\sqrt{area_{defect}}+\sqrt{area^{*}}+\sqrt{area_{0}}}}$$

The additional $\sqrt{area^{*}}$ is simply determined by [11]:

(4) $$\sqrt{area^{*}}=\sqrt{area_{0}}\frac{{\left(\Delta K_{th,eff}/\Delta K_{th,LC}\right)}^{2}}{1-{\left(\Delta K_{th,eff}/\Delta K_{th,LC}\right)}^{2}}$$

The intrinsic fatigue propagation threshold, $\Delta K_{th,eff}^{}$, can be estimated by $\Delta K_{th,eff}^{}=E\sqrt{b}$ [18]. The term $\sqrt{area^{*}}$ is introduced to fulfill the condition that $\Delta K_{th}^{}=\Delta K_{th,eff}$ for $\sqrt{area_{defect}}$ = 0. 

The intrinsic equivalent crack size $\sqrt{area_{0}}$ is given by [7]:

(5) $$\sqrt{area_{0}}=\frac{1}{\pi }{\left(\frac{\Delta K_{th,LC}}{Y\Delta \sigma_{e}}\right)}^{2}$$

where fracture is controlled by the stress intensity factor of long cracks, $\Delta K_{th,LC}$.

In the absence of large defects, the initial closure-free crack $\sqrt{area_{i}}$ can be referred as the arrested microstructurally short crack, which is given as [11]:

(6) $$\sqrt{area_{i}}=\frac{1}{\pi }{\left(\frac{\Delta K_{th,eff}}{Y\Delta \sigma_{e}}\right)}^{2}$$

**Figure 9:**
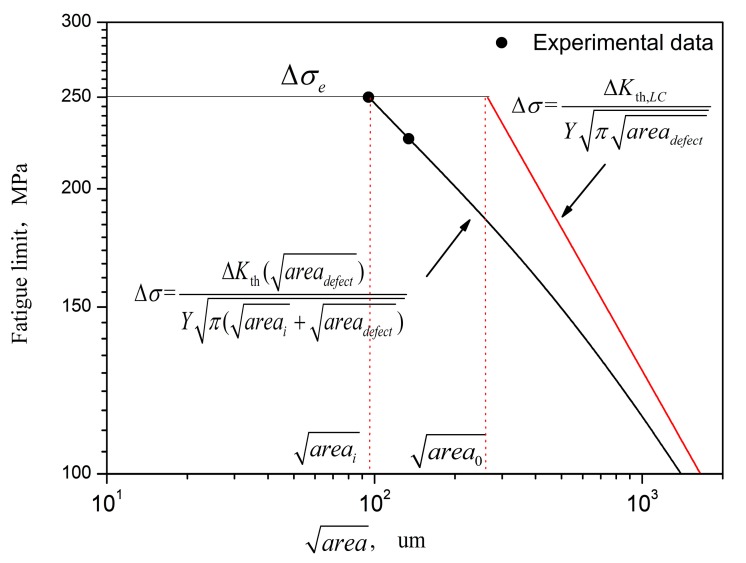
Kitagawa–Takahashi diagram of DZ4 superalloy.

The intrinsic fatigue propagation threshold of DZ4 alloy is calculated as 2.16 $\mathrm{MPa}\sqrt{m}$. The long crack propagation threshold is estimated about 3.6 $\mathrm{MPa}\sqrt{m}$ [19]. If the HIP specimens are assumed to eliminate the casting pores and have a fatigue limit Δσ_e_ (about 250 MPa), the term of $\sqrt{area_{i}}$, $\sqrt{area_{0}}$, $\sqrt{area^{*}}$ are calculated as 95 µm, 264 µm and 148.5 µm, respectively. The K–T diagram is shown in Figure 9. When considering the areas of the casting pore range from 290 to 2800 µm^2^, the mean value is 1545 µm^2^, where the equivalent size of casting pore is about 39.3 µm, the fatigue crack $\sqrt{area}$ can be about 134.3 µm, and fatigue limit of the casting specimens calculated by Equation (2) is about 226 MPa, which agrees well with the experimental data (Figure 9). This finding suggests that casting pores play a significant role in the reduction of fatigue strength, and fatigue limits of DZ4 alloy can be improved by HIP treatment, owing to the elimination of casting pores. 

### 4.3. Effect of Casting Pore on Fatigue Life

When considering that most of the total VHCF fatigue life is consumed by the crack initiation stage, fatigue life of DZ4 alloy can be estimated using a Fatigue Indicator Parameter (FIP) model that is based on the stress intensity factor close to crack initiation sites. This may reflect the relationship between Δ*K_pore_* and fatigue life (Figure 8). Steuer used the FIP model to capture the crack initiation size dependence [20]:

(7) $$FIP=\frac{\mu \sigma_{\text{a}}}{E}\left[1+k\frac{\Delta K_{defect}}{\Delta K_{th}}\right]$$

with μ the Schmid factor (equal to 0.408 for octahedral slip), parameter *k* is taken equal to 1 to obtain similar mechanical and plasticity contributions. ∆*K_defect_* can be calculated by Equation (1).

The following power-law relationship has been determined between *FIP* and the number of cycles to failure based on the fatigue data of the casting specimens:

(8) $$N_{f}=0.00161{\left(FIP\right)}^{3.302}$$

**Figure 10:**
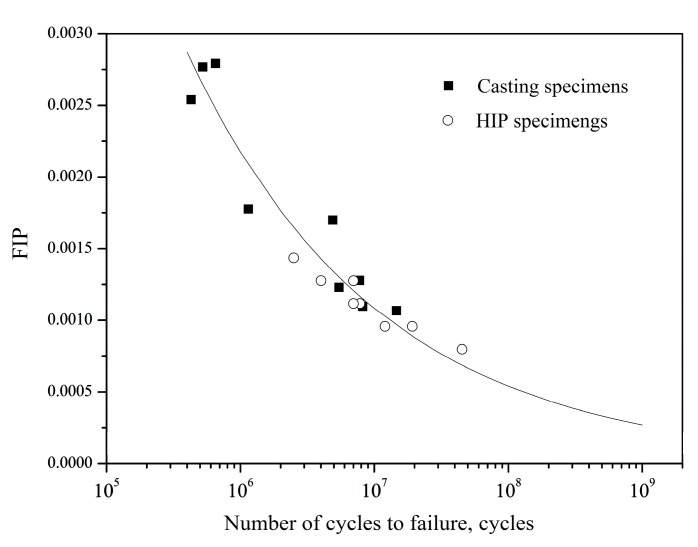
FIP (Fatigue Indicator Parameter) versus the number of cycle to failure.

**Figure 11:**
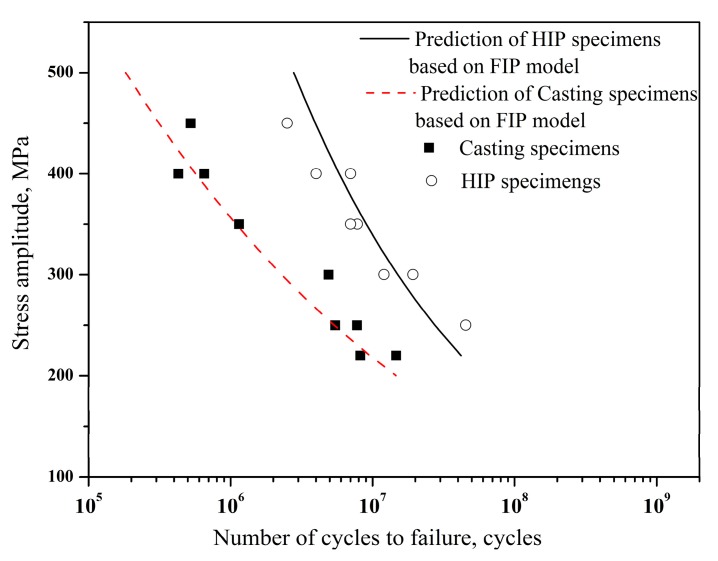
Comparison of the predicted fatigue life by using the FIP approach versus the experimental data.

The power-law and experimental data are shown in Figure 10. If the HIP specimens are assumed to eliminate the casting pores, where ∆*K_defect_* can be considered as zero, these specimens have lower value of FIP and higher fatigue life than the casting ones. The prediction of fatigue life for both casting and HIP specimens is shown in Figure 11, in which the prediction of fatigue life for casting specimens is based on the average area of casting pore (1545 µm^2^). It is suggested that the FIP model can be well used to predict the fatigue life, and the large casting defects significantly reduce the fatigue life. Thus, fatigue properties can be improved by the HIP treatment due to the elimination of the casting defects.

## 5. Conclusions

The conclusions are summarized as follows:

(1) The continuously descending S-N curves are exhibited in both the casting and the HIP specimens Fatigue fracture still occurs in both of the specimens when the number of cycles is beyond 10^7^ cycles. HIP treatment improves the fatigue properties when compared with the casting condition.
(2) The subsurface crack initiated from the casting pore in the casting specimens at low stress amplitudes, whereas fatigue crack initiated from the crystallographic facet decohesion for the HIP specimens. Considering the casting pores as initial cracks, there exists a critical stress intensity threshold ranged from 1.1 to 1.3 $\mathrm{MPa}\sqrt{m}$, below which fatigue crack may not initiate from the casting pores.
(3) Fatigue limit of the casting specimens is estimated based on a modified El Haddad model, which is well agreed with the experimental results. Fatigue life of DZ4 alloy is well predicted using the FIP model. Fatigue properties can be improved by the HIP treatment due to the elimination of casting defects.

## Figures and Tables

**Table 1:** Nominal composition of directionally solidified DZ4 Ni-base superalloy (wt %).

C	Cr	Co	Al	W	Mo	Ti	Ni
0.12	11.21	6.80	3.91	6.36	4.45	1.94	Bal.
